# One Pandemic, Two Solutions: Comparing the U.S.-China Response and Health Priorities to COVID-19 from the Perspective of “Two Types of Control”

**DOI:** 10.3390/healthcare11131848

**Published:** 2023-06-26

**Authors:** Shupeng Lyu, Chen Qian, Aaron McIntyre, Ching-Hung Lee

**Affiliations:** School of Public Policy and Administration, Xi’an Jiaotong University, Xi’an 710049, China; shupenglv@126.com (S.L.); qian_chen@stu.xjtu.edu.cn (C.Q.); ammwildhorse@gmail.com (A.M.)

**Keywords:** COVID-19, medical control, social control, zeroing policy, coexistence policy

## Abstract

After three years of global rampage, the COVID-19 epidemic, the most serious infectious disease to occur worldwide since the 1918 influenza pandemic, is nearing its end. From the global experience, medical control and social control are the two main dimensions in the prevention and control of COVID-19. From the perspective of “two types of control”, namely medical control and social control, this paper finds that the political system, economic structure, and cultural values of the United States greatly limit the government’s ability to impose social control, forcing it to adopt medical control to fight the virus in a single dimension. In contrast, China’s political system, economic structure, and cultural values allow its government to adopt stringent, extensive, and frequent social control, as well as medical control to fight the virus. This approach departs from the traditional pathway of fighting the epidemic, i.e., “infection–treatment–immunization”, thereby outpacing the evolution of the virus and controlling its spread more rapidly. This finding helps explain why the Chinese government adopted a strict “zeroing” and “dynamic zeroing” policy during the first three years, at the cost of enormous economic, social, and even political legitimacy. It was not until late 2022, when the Omicron variant with the waning virulence became prevalent, that China chose to “coexist” with the virus, thus avoiding a massive epidemic-related death. While the United States adopted a pulsed-style strategy at the beginning of the epidemic, i.e., “relaxation–suppression–relaxation–suppression”, and began to “coexist” with the virus in just one year, resulting in a large number of excess deaths associated with the epidemic. The study contributes to explaining the difference in the interplay between public health priorities and COVID-19 response strategies in China and the United States, based on the specific public health context and the perspective of “medical control” and “social control”.

## 1. Introduction

In October 2019, the Johns Hopkins Center for Health Security (JHU), the Nuclear Threat Initiative (NTI), and the Economist Intelligence Unit (EIU) jointly released the Global Health Security Index (GHS Index). Through 6 categories, 34 indicators, 85 sub-indicators, and 140 questions, this index comprehensively assessed the capability of 195 countries to prevent and mitigate epidemics and pandemics, which had been considered the most authoritative assessment and benchmarking of public health security capability. In the GHS Index, the U.S. ranked 1st, with a score of 83.5, rating as the country most capable of preventing and mitigating epidemics and pandemics, whereas China ranked 51st, with a score of 48.2, which was not only far from all major Western developed countries, but also behind countries that were less developed than China, such as Indonesia, Peru, and Vietnam. Dramatically, a few months after the index was released, the outbreak of COVID-19 spread rapidly worldwide, and was declared a global pandemic by the World Health Organization (hereafter WHO) on 11 March 2020. As of 10 May 2023, COVID-19 has spread to 235 countries and territories, with more than 6.92 million cumulative deaths worldwide [1]. Global excess deaths associated with COVID-19 between 1 January 2020 and 31 December 2021 were approximately 14.9 million [2]. COVID-19 prevention and control is a major test of the governance system and capacity of countries worldwide, and a review of the GHS Index.

Due to differences in political systems, governance ideals, and cultural values, strategies for global response to COVID-19 vary widely [3,4] and could be divided into two main types: “zeroing” policy and “coexistence” policy. China, where authoritarian governance is an important mechanism for economic development and crisis management [5], is a prime example of the “zeroing” policy. Since the very beginning of the novel coronavirus outbreak, the Communist Party of China (hereafter CPC) and the Chinese government, which were elected in a hierarchal electoral system with low public participation, adopted the “zeroing” policy out of concern for their legitimacy, and achieved zero increase in indigenous confirmed or suspected cases in April 2020. Since then, as a result of challenges caused by imported cases abroad for epidemic prevention and control, the “zeroing” policy was adjusted to the “dynamic zeroing” policy [6]. Although many countries implemented various forms of non-pharmaceutical interventions (NPIs), these might be the most stringent, extensive, and frequent in China. In the last two months before the official end of the “dynamic zeroing” policy, the livelihood issues caused by NPIs lasting three years had a high profile, and they further evolved into grievances and doubts about zero-COVID policy as tens of thousands of people on television screens cheered without masks during the FIFA World Cup Qatar 2022, resulting in small-scale protests across the country. China’s anti-epidemic strategy can be considered a success in terms of protecting people’s lives, since China managed to keep the infection, mortality, and excess mortality rates at the lowest levels in the world, albeit at great economic, social, and psychological costs. Excess deaths associated with COVID-19 between 1 January 2020 and 31 December 2021 were −52,063 [2]. After three years of hard efforts to keep COVID-19 at bay, China gained valuable windows of opportunity created by the waning virulence of the Omicron variant, mass vaccination, and improved treatment capabilities. Against this backdrop, China officially ended its “dynamic zeroing” policy, downgraded COVID-19 management, and reopened the country’s borders from 8 January 2023, shifting its focus from preventing infections toward preventing severe cases. This means that China has entered a new stage of epidemic prevention and control, i.e., “coexistence” stage. On 8 May 2023, the Joint Prevention and Control Mechanism of the State Council held a press conference and stated that China will continue to manage COVID-19 with measures against Class B infectious diseases to prevent the risk of a significant rebound and protect its people’s lives, safety and health.

Limited by the political system, economic structure, and cultural values, the U.S., with one of the most mature democratic regimes on the planet, implemented a series of non-pharmaceutical interventions during the COVID-19 outbreak, although it failed to ensure a coordinated national response and did little in the overall organization of fighting against coronavirus [7,8,9], essentially adopting a “coexistence” policy. The focus of its anti-epidemic response was to slow the spread of the virus as much as possible and “flatten the curve” of the new infections, so as to win precious time for its healthcare system to be better prepared [10]. This can be interpreted as a return to social Darwinism, which means that herd immunity against COVID-19 was gradually achieved through mass infection and vaccination, given the inability of current medical technology to completely eradicate the virus. Meanwhile, it was also motivated by the constitutional protection of individual rights, freedoms, and privacy, and the delivery of a strong economy and low unemployment, since parties were extremely sensitive to how the crisis might affect their political fortunes, especially in an election year. However, before effective therapies and vaccines were developed and the virus mutated to become more benign, most people were infected and died. The “coexistence” policy led to the widespread transmission of the virus, which not only caused significant loss of life and aggravated or even caused inequalities [11,12], but also presented an unprecedented challenge to food security, the labor market, and economic and social development [13,14,15]. At the time Trump left office in January 2021, there were more than 400,000 deaths from COVID-19 counted in the U.S. In May 2022, more than a year after Biden took office, the death toll of COVID-19 exceeded 1 million [1]. Additionally, excess deaths associated with COVID-19 between 1 January 2020 and 31 December 2021 were more than 0.93 million [2]. Despite the unprecedented vaccine push and heroic efforts of healthcare workers, the death toll of COVID-19 far surpassed that of the 1918 influenza pandemic, making it the deadliest disease in American history. On 10 April 2023, President Biden signed a House bill immediately ending the COVID-19 national emergency. Additionally, the U.S. federal public health emergency for COVID was scheduled to end on 11 May 2023. These bring a close to a slew of significant response strategies created to combat the pandemic.

The COVID-19 pandemic is the most significant health challenge that the world has confronted since the 1918 influenza pandemic. The world has paid a high price for this pandemic in two major ways: direct loss of human life and health, and indirect economic and social consequences [16,17]. On 5 May 2023, the WHO announced that the COVID-19 pandemic no longer constitutes a public health emergency of international concern (PHEIC). While this does not mean a complete end to COVID-19 as a global health threat, this officially justifies the transition from emergency mode to managing COVID-19 alongside other infectious diseases. Given that many countries have already relaxed measures to combat COVID-19, it is urgent to ensure that countries do not turn their back on learning the lessons of COVID-19, and strengthen their preparedness for future pandemics. As two of the most powerful countries, China and the U.S. have shown completely different endeavors and effects of epidemic prevention and control. Against the backdrop of increasingly fierce and unpredictable competition among major powers [18] and the world’s accelerating changes unseen in a century, such differences signify a potential international order transition and the reform of the global governance system. In particular, the interplay between the public health priorities and COVID-19 response strategies embedded therein might profoundly influence future strategies and modes for combating epidemics and pandemics within and beyond these two countries. However, few studies conducted the systematic comparison and mechanism analysis of anti-epidemic policies and measures between the two countries over different periods. To that end, this study will attempt to analyze the different anti-epidemic modes adopted by China and the U.S. from the perspective of “two types of control”.

The paper is organized as follows. After an introduction of the definitions and influencing factors of two types of control in Section 2, the differences in two types of control between China and the U.S. are elaborated in Section 3, the control strategies for COVID-19 are discussed in Section 4, and conclusions are provided in Section 5.

## 2. Materials and Methods

### 2.1. Study Area and Analysis Method

Taking China and the U.S. as examples, this study analyzes the COVID-19 response strategies in both countries from the beginning of the COVID-19 pandemic to the present, based on a time slicing approach. The stage division of the practice to prevent and control COVID-19 in the two countries is based on the type and characteristics of the virus and the basic orientation of the response strategy at that time. Additionally, based on relevant data published by WHO, Johns Hopkins Coronavirus Resource Center, Organisation for Economic Co-operation and Development (OECD), Centers for Medicare and Medicaid Services, and National Health Commission of the People’s Republic of China, this study discusses the health and social consequences of the COVID-19 response strategies in both countries.

### 2.2. Definitions and Influencing Factors of Two Types of Control

Newly emerging and re-emerging infectious diseases have been threatening human society since the neolithic revolution [19]. The interactions between infectious agents, hosts, and the social environment may cause epidemics or even pandemics. That is, although infectious diseases themselves are caused by pathogens, their interactions with hosts and the social environment are the underlying or contributing causes of epidemics and pandemics. In recorded human history, there have been several pandemic explosions, such as the 1918 influenza pandemic. COVID-19, first officially identified in China, is the latest example of an unprecedented pandemic. It can be said that human society has entered the modern epidemic or pandemic era. The wide-ranging and devastating impacts of epidemics and pandemics provide a compelling rationale for their prevention and control, whether it is immediate or long-term. Based on past successes in controlling infectious disease outbreaks, this study divides the responses involved into two aspects: medical control and social control.

#### 2.2.1. Medical Control

For this study, medical control refers to medical countermeasures in the event of infectious disease outbreaks, involving diagnosis, treatment, and prevention. It can include public health surveillance and early warning of infectious disease outbreaks, the development of diagnostic tools, drugs, therapies, and vaccines, the sufficient supply of healthcare workers and medical resources, etc.

The influencing factors of medical control can be summarized as the following four aspects. The first is medical technology, which is a broad discipline and significantly alters the healthcare environment. Medical technology, such as DNA sequencing technology and mRNA technology, can help diagnose and treat patients, prevent infections, and reduce morbidity and mortality in the face of infectious disease outbreaks. The second is medical resources, which refers to the facilities, services, and products used in healthcare delivery, such as hospital beds and healthcare workers. The abundance and efficient allocation of medical resources can help ensure people’s right to medical care, alleviate social panic, and promote the health and well-being of individuals in the event of infectious disease outbreaks. The third is health expenditure, which includes all expenditures for the provision of healthcare services, emergency aid designated for health, etc. Although existing studies have revealed mixed impacts related to health expenditure on health metrics [20,21], increasing health expenditure is always linked to public health performance improvement and positive effects on overall well-being in the event of infectious disease outbreaks. The fourth is the primary healthcare system, which is the first line of defense against public health events. As a widely recognized approach to bringing healthcare services closer to individuals, families, and communities, the primary healthcare system is conducive to improving the resilience of national healthcare systems to prepare for, respond to, and recover from infectious disease outbreaks.

#### 2.2.2. Social Control

For this study, social control refers to transmission-based interventions and supportive actions in the event of infectious disease outbreaks, which is the additional and critical strategy for the national response to outbreaks used when the spread of infections cannot be prevented or stopped by medical control alone. Social control, including contact tracing, quarantine and isolation, social distancing, and strengthening of border healthcare systems at ports of entry, aims to contain and prevent the transmission of viruses across borders, within communities, and among susceptible persons, support essential supplies for outbreak response, maintain essential health services and keep healthcare systems from being overwhelmed.

The influencing factors of social control can be summarized as the following four aspects. The first is the political system, which refers to the set of institutions that make and enforce laws, distribute power, regulate social activities, etc., that involves not only the constituent elements of government and the country, but also the process of how they function. The political system plays an important role in releasing and implementing costly social control measures, setting priorities and allocating scarce resources, and strengthening community participation during infectious disease outbreaks. The second is the economic system, which refers to the organized way in which a country has arranged its material provisioning, and significantly influences the values and political structure of the country. The economic system, involving the interaction of all entities in the economy, determines the ability of a country to mobilize various sectors and professionals, overcome market failures, and secure supply chains of critical items needed to address a wide range of health and social issues in the event of infectious disease outbreaks. The third is the public health emergency management system, which refers to a comprehensive command and coordination system that includes emergency organizational structure, emergency subjects, emergency mechanisms, and emergency resources. This system is on the frontline of providing public health interventions involving prevention, preparedness, response, recovery, and reducing the impacts on public health and social stability before, during, and after infectious disease outbreaks. The fourth is culture, which is a broad term, that includes social norms, values, beliefs, etc. Culture plays a vital role in the decision-making process of government, public perception of collective actions, and individuals’ compliance with government involvement in the face of infectious disease outbreaks [22].

#### 2.2.3. Control Strategies for Different Types of Infectious Disease Outbreaks

**Figure 1:**
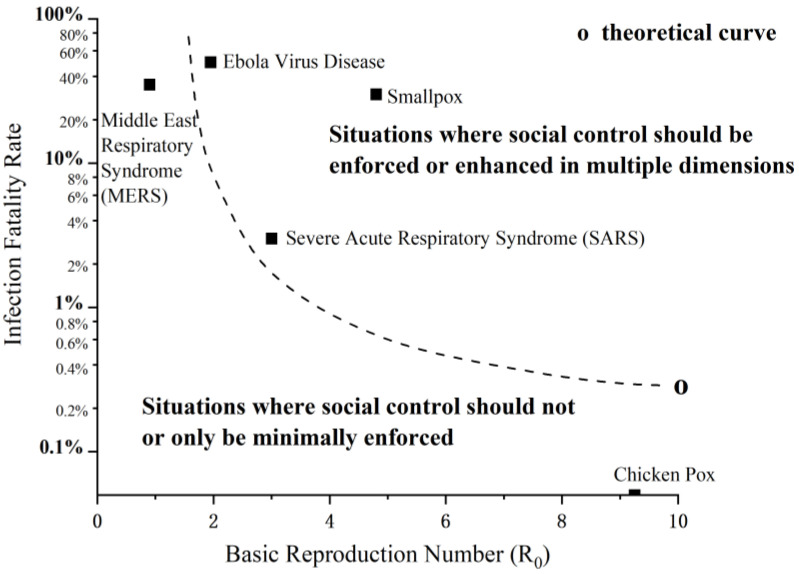
Theoretical modes for the prevention and control of infectious disease outbreaks. Data source: WHO and references [25,26,27,28,29].

As biological and social phenomena, infectious disease outbreaks are inevitably shaped and changed by human responses from the very beginning to the very end. Following the history of experiences in the prevention and control of infectious disease outbreaks, medical control and social control measures have constituted the responses to these threats, and accounted for the remarkable progress in containing them [23]. In the prevention and control of infectious disease outbreaks, the role of medical control and social control is fundamental and supportive, respectively. Despite advances in modern medicine, the primary function of medical control is palliation, i.e., relieving pain, reducing disability, and prolonging death in some cases of medical care [24], which is limited by medical technologies and medical resources. When medical control is temporarily difficult to contain an outbreak, a close look at alternative or complementary solutions, i.e., social control, is required to discover suitable modes of combinations between them. While there is a risk of not adopting or relaxing social control in the face of a significant biological threat, there is also a risk of misjudging the threat and adopting social control too hastily or too early, so that these restrictions cannot be properly taken back. In other words, there is a problem with the applicable situation in social control. The characteristics of viruses and previous control experiences are illustrated in Figure 1. For infectious disease outbreaks, such as chickenpox, which have an extremely high infection rate and an extremely low infection fatality rate, it can be completely prevented and controlled by medical control, such as drugs and vaccines, without crushing impacts on public health, economy, and society. For infectious disease outbreaks such as MERS, which have an extremely low infection rate and an extremely high infection fatality rate, the virus will disappear rapidly due to the death of the host, making it difficult to spread widely among the general population. Therefore, the prevention and control of such outbreaks should also focus on medical control without excessive social control. For infectious disease outbreaks such as Ebola, which have a high infection rate and an extremely high infection fatality rate, the virus can spread further and faster by killing, maiming, or severely harming fewer confirmed cases. For infectious disease outbreaks such as SARS, which have a high infection rate and a low infection fatality rate, its characteristics elevate the virus from an occasional nuisance to one capable of causing an epidemic or pandemic. Thus, the prevention and control of such outbreaks require the intervention of both medical control and social control. It should be noted that the dotted line in the figure represents only a set of approximate, rather than exact, values, and countries may differ because of their capabilities in two types of control.

## 3. Results

### 3.1. Differences in Two Types of Control between China and the U.S.

#### 3.1.1. Differences in Medical Control

Firstly, the U.S. is a global leader in medical science and technology by a wide margin. In terms of new drugs and medical devices, two commonly used indicators of the level of medical technology, the U.S. accounted for 56% and 54% of the global new drugs and biologics market, respectively, in 2019 and 2020, while China’s market shares in these two years were all 12% [30,31]. At the same time, the U.S. accounts for about 40% of the global medical device market, and has most of the world’s top medical device companies, such as Medtronic, Johnson & Johnson, Baxter, etc. Even with a population four times that of the U.S., China accounts for about 21% of the global medical device market, and has a handful of the world’s top medical device companies, such as Mindray Medical. Secondly, the U.S. has more abundant medical resources than China. According to data released by Organisation for Economic Co-operation and Development (OECD), in 2019 and 2020, the number of doctors per 1000 inhabitants was 2.64 and 2.90, the number of nurses per 1000 inhabitants was 11.97 and 11.83, and the number of intensive care unit beds per 100,000 inhabitants was 25.8 and 21.6 in the U.S. In these two years, the number of doctors per 1000 inhabitants was 2.24 and 2.36, the number of nurses per 1000 inhabitants was 3.10 and 3.27, and the number of intensive care unit beds per 100,000 inhabitants was 4.05 and 4.50 in China [32]. Thirdly, the health expenditure in the U.S. is much higher than in China. Specifically, in 2019 and 2020, total health expenditure in the U.S. accounted for 17.6% and 19.7% of GDP [33], while in China accounted for 6.6% and 7.1% of GDP [34]. In these two years, per capita health expenditure in the U.S. was about 16.7 and 16.6 times higher than that in China. Finally, the healthcare system in the U.S. is highly structured, hierarchical, and efficient, involving high-quality primary healthcare [35]. Insured individuals tend to seek care from a primary care provider, who will examine and evaluate the patient first and decide whether a referral is needed or not. Although uninsured individuals usually do not have a regular primary care provider, they may not seek care through community health centers or hospitals until an emergency occurs, because of out-of-pocket costs [36]. There is recent evidence of a decrease in problem-based primary care visits in the U.S. [37]. In addition to other mechanisms, more non-face-to-face care may largely explain this decline, which also results in fewer follow-ups and unneeded appointments [38]. While China has established a three-tier healthcare delivery system in both rural and urban areas and achieved remarkable results in strengthening it, some serious challenges remain for this system in providing essential health services and generalist clinical care [39]. Due to various factors, such as rapid income increase, population aging, lack of formal referral mechanisms, and weak primary healthcare system, multilevel referrals in China are practically unrealized [40,41]. A common phenomenon in China is that patients tend to bypass the primary healthcare system and seek more specialized consultation and care from secondary and tertiary healthcare facilities, resulting in extremely high patient flow to large hospitals [42]. Thus, in the event of infectious disease outbreaks, the healthcare system in the U.S. may not only minimize the risk of virus transmission, but also prevent the system from being overwhelmed by eliminating nonessential in-person visits, differentiating patients early, and initiating the most appropriate care pathway, as compared to China.

In summary, medical control in the U.S. may be superior to that of China for responding to infectious disease outbreaks.

#### 3.1.2. Differences in Social Control

Firstly, China is an authoritarian country characterized by centralized leadership, top-down rule, strong bureaucratic mobilization capacity, and institutionalized but restricted participation and deliberation [43]. The CPC is the sole political party in power, and its leadership of the country is mainly reflected in political, economic, and ideological matters. Only the Party Central Committee has the authority to decide on major national issues. Party organizations of various departments and localities can make suggestions to the Central Committee, but they must not make unauthorized decisions or publicize their proposals. The U.S. is a constitution-based federal republic, characterized by a representative democracy, separation of powers, majority rules, and a highly diverse population. The federal and state governments are divided into three branches, i.e., the legislative branch, the judicial branch, and the executive branch. The checks and balances system provides each branch with individual powers to check the others and prevent any one branch from becoming too powerful. In the U.S., politics and governments are dominated by a two-party system, i.e., the Republicans and the Democrats, in which many factions are struggling for power and shifting coalitions are formed. Organization and control may be relatively strong at the local level, but this is much weaker at the state level and almost invalid at the national level. Driven by these characteristics, many divisions have been created within politics, leading to a lack of timely and unified action to address pressing issues, which are even prone to become bargaining chips for politicians. Thus, in the face of infectious disease outbreaks, China may be able to release and implement social control measures, such as lockdowns and massive screening, more quickly, uniformly, and rigorously than the U.S. Secondly, China operates as a socialist market economy characterized by state-owned enterprises and public ownership. Although the market plays a fundamental and decisive role in resource allocation with a broader scope and, to a greater extent, adhering to the leadership of the CPC and government in economic work, and performing the role of the “visible hand” in a better way, is always the foundation of the defining property of the socialist market economy. The U.S. operates as a mixed economy that exhibits characteristics of both capitalism and socialism. It not only embraces the free market in the use of capital, but also allows the governments to partially control the economy with regulatory restrictions. In general, China may be able to intervene in economic affairs to a greater extent and on a broader scale than the U.S. In the event of infectious disease outbreaks, compared to the U.S., China is not only able to suspend non-essential industrial and commercial production activities and prohibit group gatherings in public places quickly and indefinitely, such as closing restaurants and supermarkets by administrative means, but also open green channels and speed up the production, transport, and delivery of critical supplies, and any other activities relevant to fight against the outbreak. China can even provide completely free medical services to patients through state financial support, as it did in response to the SARS outbreak, so that each patient can be treated and all possible transmission can be prevented. Thirdly, boosted by the SARS outbreak, China has gradually established and improved its public health emergency management system, with “one case, three systems” as the core framework. It takes the CPC Central Committee as the leading core, and the State Council as the highest executive entity, following the principle of unified leadership, comprehensive coordination, graded responsibility, and territorial management. After experiencing many twists and turns, as well as achieving remarkable results, it is working toward a more digital, equitable, and people-centered approach [44,45]. The U.S. has established a public health emergency management system with a vertical hierarchy of “federal–state–local”, and a horizontal division of labor between governments, private sectors, and volunteer groups, resulting in poor emergency coordination among different governments and departments. State and local governments have a high degree of autonomy in public health governance, and have the power to decide on prevention and control measures in their regions, which cannot be directly interfered with by the federal government. Although both countries have established an advanced management system to respond to public health emergencies, compared to the U.S., China’s public health emergency management system is more conducive to achieving cross-sector, cross-level, and cross-regional coordination, as well as forming a national disaster relief system, joint prevention and control mechanism, and pairing assistance mechanism to combat the outbreak. Finally, Chinese people are deeply influenced by Confucianism, and have a strong collective spirit and a sense of identity and reverence for national authority. At the same time, several cross-country polls and studies revealed that China enjoyed a high level of political trust, especially in the central government [46]. Individualism became part of the core American ideology by the 19th century, and was baked into American culture, which had a tremendous impact on social theory and political philosophy. Not only do Americans place a high value on individuality, independence, rights, and the protection of personal privacy, but they also have a low level of trust in government, especially the federal government, and a negative attitude toward authority. As a result, compared to the U.S., China’s cultural beliefs and values make social control an easier task in the event of infectious disease outbreaks.

In summary, social control in China may be superior to that of the U.S. for responding to infectious disease outbreaks.

#### 3.1.3. Control Strategies for Different Types of Infectious Disease Outbreaks in China and the U.S.

**Figure 2:**
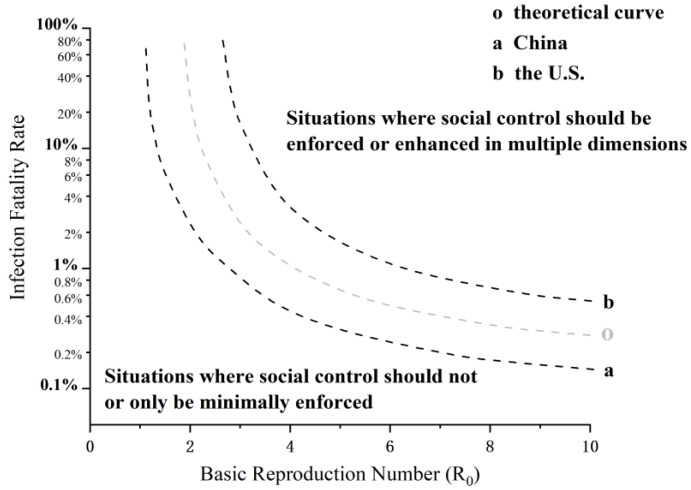
Actual modes for the prevention and control of infectious disease outbreaks in China and the U.S.

Given the differences in two types of control between China and the U.S., the actual curve for the prevention and control of infectious disease outbreaks in China may lie below the theoretical curve, while it may lie above the theoretical curve in the U.S., as shown in Figure 2. The range of the basic reproduction number and infection fatality rate of COVID-19 may lie exactly between the actual curves of the two countries, leading to the SARS-like response in China and the influenza-like response in the U.S.

### 3.2. Control Strategies for COVID-19 in China and the U.S.

#### 3.2.1. Control Strategies for COVID-19 in China

**Figure 3:**
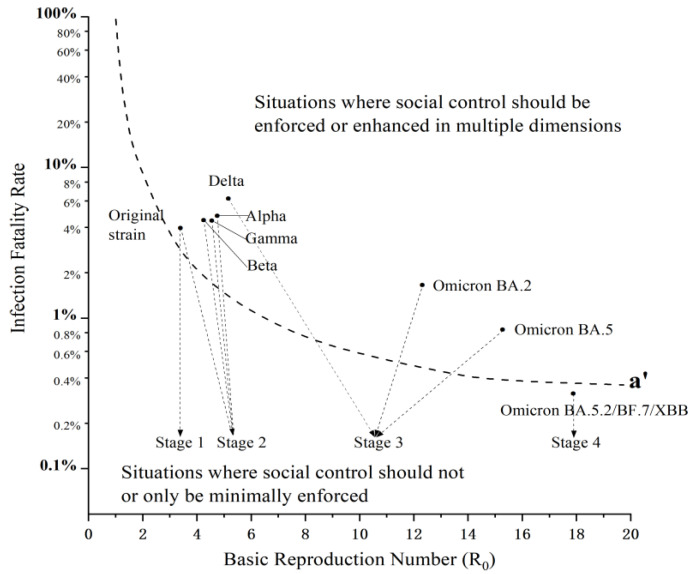
Dominant strains of SARS-CoV-2 and control strategies in China.

Based on the dynamic adjustment of anti-epidemic strategies, China’s practice to prevent and control COVID-19 can be roughly divided into four stages, as shown in Figure 3.

The first stage was the rapid containment stage of a public health emergency (from December 2019 to March 2020). At this stage, China was faced with the original strain of SARS-CoV-2, which was later found to have a basic reproduction number between 2.24 and 3.58 [47] and an infection fatality rate between 1.54% and 3.4% [48]. Given the rapid spread and severity of the outbreak that overwhelmed diagnostic and healthcare capacities, China timely adopted extensive and aggressive social control measures, successfully broke the chains of transmission at community and household levels, and achieved decisive results in the defensive battles of Wuhan and Hubei in about three months. In April 2020, the Chinese mainland reported zero increase in indigenous COVID-19 cases.

In terms of medical control, China conducted scientific research on diagnosis, treatment, and laboratory testing, and built a treatment system of integrated traditional Chinese medicine and Western medicine. Testing kits were rapidly developed, and the National Health Commission (hereafter NHC) distributed nucleic acid reagents for test kits to various health departments. Based on specific symptoms, “three drugs, three formulas” and other antiviral drugs and formulas were created to treat patients. Several beneficial therapeutic approaches, such as stem cell-based therapy and convalescent plasma treatment, were developed to treat COVID-19 and its complications. During this stage, NHC published seven editions of diagnosis and treatment protocol for COVID-19. In addition, China established a medical insurance reimbursement policy, which indicated that the cost of medicines and medical services for treating COVID-19 would be entirely covered by the insurance fund.

In terms of social control, on 23 January 2020, China decided to close outbound traffic from Wuhan, i.e., lockdown, the largest transportation hub in the inland, with a population of 12 million, followed by other cities in the Hubei province. To resolutely curb the spread of the virus, China implemented travel restrictions and social distancing measures, such as school and entertainment venue closures and the prohibition on group gatherings, and confined 1.4 billion people in their homes for almost three weeks. The government issued instructions for industrial plants to churn out protective gear, ventilators, air disinfectant machines, and hemodialysis machines, so as to ensure that there was no shortage of medical equipment. Meanwhile, grassroots organizations across the country screened four categories of people, including confirmed cases and their close contacts, suspected cases, and febrile patients, through visits, surveys, and big data analysis, and implemented classified management in designated medical institutions. In addition, China mobilized hundreds of medical teams, tens of thousands of healthcare workers, and a large number of medical supplies from various regions to support Wuhan and Hubei.

The second stage was the exploration stage of regular epidemic prevention and control (from April 2020 to April 2021). At this stage, China experienced many outbreaks, with local transmission caused by imported SARS-CoV-2, including the original strain and Alpha, Beta, and Gamma variants. These three variants were 43%–90% [49], 25%–60%, and 38%–60% [50,51] more transmissible than the original strain, and the hazard of death associated with them was 61%–67% [52,53], 26%–74% [54], and 24%–54% [55] higher than with the original strain, respectively. China adjusted its general response strategy to “preventing the coronavirus from entering the country and stemming its domestic resurgence”, aligning with changes in the epidemic situation. China combined medical control measures with social control measures to combat the epidemic, with the former focusing on nucleic acid tests and scientific research and development, and the latter focusing on closed-loop management and small-scale lockdown, basically containing sporadic outbreaks within 2–3 incubation periods.

In terms of medical control, China was committed to strengthening diagnostic schemes and tools research and development, and identifying the infected as soon as possible through active surveillance of key populations and sentinel surveillance of fever clinics. At the same time, China vigorously strengthened the development, approval, and regulatory oversight of antiviral drugs and vaccines, and promoted research on virus mutations and immunization strategies. On 31 December 2020, China’s National Medical Products Administration granted conditional market approval to its first COVID-19 vaccine, and since then, China fully launched vaccination efforts.

In terms of social control, China strictly implemented closed-loop management in air transportation, border crossings, and other important links, realized the joint prevention of people, objects, and the environment, and insisted on the combination of regular prevention and control measures and localized emergency response measures. The localities dynamically adjusted the epidemic risk level, which was divided into three tiers, i.e., high risk, medium risk, and low risk, and matched them with differentiated management measures. The NHC issued and revised guidelines that outright ordered its residents to don masks in public. Meanwhile, relying on the national integrated government service platform, China promoted the mutual recognition of “health codes” in various regions, and timely shared information to the “health code” database, such as nucleic acid test results and travel and residence history in risk areas, so as to achieve safe and orderly travel and rapid and accurate epidemiological survey. Although digital solutions were considered as an alternative to lockdowns, they may have potentially adverse effects on data governance and society in the long run [56].

The third stage was the “dynamic zeroing” stage, characterized by science and precision (from May 2021 to November 2022). From May 2021 to February 2022, the Delta variant was the dominant strain of the coronavirus in China. It was 97%, 55%, 60%, and 34% [51] more transmissible than the original strain and Alpha, Beta, and Gamma variants, respectively, and the hazard of death associated with it was 115% [57], 70%, 55%, and 120% [58] higher than with the original strain and Alpha, Beta, and Gamma variants, respectively. From March 2022 to the present, the Omicron variant, identified as being significantly more transmissible, replaced the Delta variant as the dominant strain in China. The Omicron subvariant BA.2 was the dominant strain, with a basic reproduction number of about 12.3 [59,60] and an infection fatality rate of about 0.63% [61,62] from March to May 2022. In May 2022, the Omicron subvariant BA.5 replaced BA.2 as the dominant strain, which was the most transmissible strain at that time, with a strong immunity evasion capability, but its surge proved much milder than the previous waves, in terms of deaths and hospitalizations [63]. In response to the development of the epidemic situation, and given that the population immunity barrier was not yet established, China adopted a new policy called “dynamic zeroing”, and aimed to control the spread of the disease at a lower cost and in a shorter time, which was drawn on its previous experiences in fighting against dozens of outbreaks. The most important purpose was to minimize the impacts of epidemic prevention and control on the economy, society, and normal lives, and strike a balance between epidemic prevention and control and economic and social development. At this stage, China further optimized medical control measures and social control measures in the face of high transmission of variants, with the former focusing on the nucleic acid test and vaccination, and the latter focusing on the precise epidemiological investigation and zero community transmission, basically containing sporadic outbreaks within one incubation period.

In terms of medical control, China promoted the improvement of the regular mechanism for the nucleic acid test, and established nucleic acid sampling circles with a 15 min walk in provincial capitals, port cities, and cities with a population of 10 million. Once the infected were discovered, healthcare workers conducted one or more rounds of mass testing. China added antigen detection as a complement to the nucleic acid test, and strengthened the early detection of outbreaks through the model of “antigen screening and nucleic acid diagnosis”. To avoid the overwhelming strain on medical resources, China improved the classified admission mechanism, and implemented centralized isolation and management for asymptomatic cases and mild cases, who would be transferred to designated hospitals for treatment after aggravation. Meanwhile, China planned and deployed designated hospitals, sub-designated hospitals, etc., to enhance the capacity of isolation and treatment. In addition, China continued to promote the development and approval of antiviral drugs, expanded the scope of vaccination to people over 3 years old, and promoted vaccination and booster shots.

In terms of social control, China precisely divided close contacts, secondary contacts, and general contacts, and accurately delineated the scope of lockdown, control, and precaution zones to the smallest unit. When there was a local recurrence, governments moved immediately to seal residential compounds, urban districts, or even entire cities, and implemented classified management for different zones and populations. China took full advantage of the golden 24 h after each outbreak to find and control potentially infected individuals by mobilizing extensive community involvement, applying new technologies, and strengthening financial support. At the same time, a widespread quarantine was quickly imposed to stem community transmission of the virus. The quarantine at home or designated sites was often severely restricted to ensure compliance. Furthermore, China tightened the mask requirements and ordered the public to wear masks in indoor venues, public transport, and crowded outdoor areas.

The fourth stage was the “coexistence” stage with COVID-19 (from December 2022 to the present). BA.5.2 and BF.7, sub-lineages of the Omicron subvariant BA.5, and Omicron XBB variant, were the dominant strains of the coronavirus in China at this stage. They were identified to have more immune escape capability, higher transmission rate, and lower risk of causing severe illness and death, than previous strains [64]. Numerous scientific studies showed that COVID-19 vaccines retained the strong ability to prevent severe illness and death caused by Omicron and its sub-variants for a certain period, despite waning vaccine effectiveness over time [65,66]. As of 11 November 2022, 90.26% of the population had been fully vaccinated in China. Meanwhile, China’s testing, diagnosis, and treatment have been steadily rising. Against this backdrop, China officially ended its “dynamic zeroing” policy and made several positive and bold adjustments to the COVID-19 response, including 20 optimized measures in November 2022 and 10 new measures in December 2022, which meant that China officially moved towards reopening. China lifted social control measures on a wide scale, and shifted the focus of its anti-epidemic efforts from curbing infection to medical treatment. As of February 2023, China has established sound herd immunity after resisting the latest major outbreak. Yet, China stands a high probability of a second wave of COVID-19 infections in the near future, due to the declining antibody levels in the population and the massive movement of people during the May Day holiday.

In terms of medical control, China urged cases with mild or no symptoms to recover at home, and concentrated medical resources on protecting vulnerable groups and treating severe cases. China strived to strengthen its medical capacity in an all-round way for predicted peak and possible exit waves, such as increasing the number of intensive care beds, applying internet hospitals, enhancing medical services in rural areas, producing and distributing COVID-19-related medicines, strengthening its primary healthcare system, and promoting the tiered system of diagnosis and treatment. Meanwhile, China spared no effort to improve the vaccination rate, especially for those aged between 60 to 79 years old, and those 80 or above, by setting up special and temporary vaccination sites and deploying mobile vaccination vehicles. In addition, China is closely monitoring the situation and detecting potential emerging variants.

In terms of social control, China downgraded the management of COVID-19 from top-level Class A to Class B, according to the Law of the People’s Republic of China on Prevention and Treatment of Infectious Diseases. China also removed COVID-19 from quarantinable infectious disease management, according to the Frontier Health and Quarantine Law of the People’s Republic of China. Authorities dropped almost all social control measures, such as quarantine, social distancing measures, and contact tracing, and focused on protecting health and preventing severe cases. At the same time, quarantinable infectious disease control measures were no longer implemented for imported goods or inbound persons, and outbound tourism and passenger entry and exit at sea and land ports were restored. Furthermore, China changed the official Chinese term for COVID-19 from “novel coronavirus pneumonia” to “novel coronavirus infection”.

#### 3.2.2. Control Strategies for COVID-19 in the U.S.

**Figure 4:**
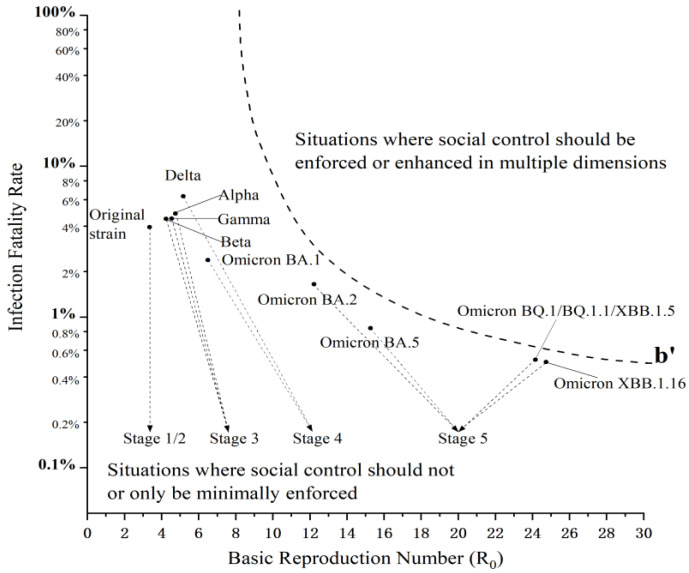
Dominant strains of SARS-CoV-2 and control strategies in the U.S.

Based on the dynamic adjustment of anti-epidemic strategies, the practice of the U.S. to prevent and control COVID-19 can be roughly divided into five stages, as shown in Figure 4.

The first stage was the emergency suppression stage (from late February to April 2020). The original strain of SARS-CoV-2 was the dominant strain of the coronavirus in the U.S. at this stage. The U.S. missed opportunities to curb COVID-19 due to the Trump administration’s underestimation of this crisis, and inaction or partial measures at the very beginning of the outbreak. According to a report published in Science on 28 February 2020, only 459 tests for novel coronavirus had been completed in the U.S. as of that time [67]. Virus tests in the U.S. came to a near halt during an extremely critical period for epidemic prevention and control, which continued to cripple its responses to this epidemic for months. Faced with the rapid community outbreak of COVID-19, the Trump administration declared a national emergency on 13 March 2020, and began to combat the coronavirus by adopting social control measures, such as stay-at-home orders, as well as medical control measures, such as mass nucleic acid tests.

In terms of medical control, the Food and Drug Administration (hereafter FDA) eased restrictions on the development and use of other test kits and simplified the test approval process in late February 2020. The Centers for Disease Control and Prevention (hereafter CDC) formally removed test restrictions and made clear that “any American can be tested” for the coronavirus on March 4. Trump exercised his authority to ramp up the production of much-needed medical supplies and signed several memoranda and executive orders to provide aid to states. The drive-through testing sites were rolled out across the country to improve surveillance and relieve the burden on hospitals. To fill the health workforce gap, some states passed legal exemptions that temporarily removed some practice requirements of medical personnel, and issued emergency nursing and medical licenses. Additionally, many states allowed healthcare workers who tested positive for COVID-19, but were asymptomatic, to continue working. Some routine healthcare services were suspended, and excess capacity was used for the treatment of COVID-19. Meanwhile, some parks, convention centers, and empty fields were transformed into emergency field hospitals, and many extra beds were available to deal with a looming shortage of hospital beds. In addition, the FDA and CDC greatly simplified the approval process for antiviral drugs and test kits, and spared no effort to develop vaccines and discover effective therapies.

In terms of social control, Trump issued proclamations that banned the entry of non-U.S. citizens who were from or recently had been in China, Iran, or certain European countries, and that American citizens, legal permanent residents, and their immediate families returning from these countries must travel through designated airports and receive an enhanced entry screening, and self-quarantine for 14 days after arrival. The CDC issued a No Sail Order for cruise ships, suspending operation in U.S. waters. Trump issued new guidelines on 16 March that called for people to avoid social gatherings of more than 10 people and stay away from bars and restaurants, and restricted discretionary travel. Trump also called for governors to implement closures on venues where people usually gathered in states with evidence of community transmission, such as schools, bars, and restaurants. As of the end of March, 39 states had banned gatherings, 44 states had closed bars and restaurants for dine-in seating, and 47 states had forced school closures. Additionally, 29 states had issued stay-at-home or shelter-in-place orders designed to limit residents’ movements to essential activities [68], but their enforcement varied widely, and they were ineffective at generating sustained behavior change [69,70]. This resulted in many dissatisfied people, some of whom organized and participated in protests against the government’s orders in several states. In April 2020, the CDC recommended that people wear cloth face coverings in public places.

The second stage was the tentative reopening stage (from May to December 2020). The original strain of SARS-CoV-2 remained the dominant strain of the coronavirus in the U.S. at this stage. Against the backdrop of the downward trend in the number of COVID-19 infections, the U.S. began to lift social control measures, such as stay-at-home orders, and shift the focus of its anti-epidemic efforts to medical control measures, such as vaccine research and development. At the same time, the Trump administration advocated a comeback from the coronavirus’ grip, and decided to reopen the U.S. economy regardless of experts’ warnings.

In terms of medical control, the Trump administration launched Operation Warp Speed (OWS) in May 2020 to accelerate the development, manufacturing, and distribution of COVID-19 vaccines, therapeutics, and diagnostics. The U.S. developed antivirals and vaccines, and eliminated the bottlenecks involved, at an unprecedented speed, to mitigate the direct and potential impacts of COVID-19. Specifically, the FDA authorized the antiviral drug remdesivir for emergency use in treating coronavirus cases in May 2020, and approved it for the treatment of suspected or laboratory-confirmed COVID-19 in adults and pediatric patients hospitalized with severe disease in October 2020, which was the first drug approved to treat COVID-19. In August 2020, the FDA issued an emergency use authorization (hereafter EUA) for the emergency use of COVID-19 convalescent plasma for the treatment of hospitalized patients with COVID-19. In December 2020, the FDA authorized the first coronavirus vaccine for emergency use in people 16 years of age and older, known as the Pfizer-BioNTech COVID-19 Vaccine. Vaccination officially began on 14 December 2020, with priority for healthcare workers and nursing home staffers. Subsequently, the FDA issued an EUA for a COVID-19 vaccine made by Moderna, Inc., a biotechnology company based in Massachusetts. In addition, the Trump administration deployed doctors, nurses, and other healthcare workers to coronavirus hot zones, ramped up production of critical medical supplies, and allowed non-hospital spaces to be used for treatment and quarantine.

In terms of social control, the Trump administration released federal guidelines urging states to reopen the economy amid the coronavirus pandemic. Several states began to resume catering, construction, and manufacturing while they did not yet have the capabilities of adequate tests, isolation, and contact tracing, leading to more spikes in cases. States urged or made masks mandatory in public places, but enforcement lagged. Trump had been holding campaign rallies, which were suspended after declaring COVID-19 a national emergency on 13 March but resumed in late June, across the country, and hosting large events at the White House. A federal leadership vacuum led to a patchwork of efforts nationwide, yet decades of underinvestment resulted in a decline in the ability of states and local public health departments to respond to the epidemic [71]. A surge in cases during the summer led some states to slow their reopening. As cases decreased in the late summer and early fall, the U.S. continued to increase reopening efforts. A resurgence in cases during the winter prompted some states to slow their reopening again. Their responses to the epidemic lacked continuity and consistency, and fell along political lines. In September 2020, the Trump administration halted its enhanced entry health screening for passengers at 15 designated airports, and only implemented it for passengers from certain countries.

The third stage was the slight tightening stage (from January to April 2021). At this stage, several variants such as Alpha, Beta, and Gamma emerged in the U.S. Immediately after taking office, Biden rolled out a national strategy to combat the epidemic, not only adopting medical control measures, but also further tightening social control measures such as mask mandates. New confirmed cases trended down, since hitting a peak in early January 2021.

In terms of medical control, the FDA issued an EUA for the third COVID-19 vaccine in February 2021, which was developed by the Janssen Pharmaceutical Companies of Johnson & Johnson. The Biden administration invoked the Defense Production Act to expand medical supplies production and ensure equitable distribution to states. Meanwhile, the Biden administration was committed to increasing vaccination rates by creating more places for Americans to get vaccinated, launching a federally supported website to find vaccines, and deploying many active-duty troops to support vaccination efforts. The U.S. achieved Biden’s goal of administering 200 million shots within his 100th day in office, but fell short of the goal of vaccinating 70% of adults with at least one dose by 4 July (Independence Day). Although the safety and efficacy of vaccines to prevent infection with SARS-CoV-2 were supported by clinical and scientific data, vaccine hesitancy remained a significant problem in the U.S. [72,73].

In terms of social control, Biden signed several executive orders about mask-wearing requirements in January 2021, including requiring travelers to wear masks on public transportation and at transportation hubs, requiring on-duty or on-site federal employees, on-site federal contractors, and other individuals to wear masks in federal buildings and on federal lands, challenging all Americans to wear masks for the next 100 days, etc. The CDC released a mandate that required international travelers to obtain a negative coronavirus test at least three days before their flight, and quarantine for seven days after arriving in the U.S. Biden rejected Trump’s order to lift travel bans on the UK, Europe, and Brazil, which started in March 2020, and continued to maintain them. Biden also banned the entry of most non-U.S. citizens traveling from South Africa, where a new strain of the coronavirus was identified. Biden signed the USD 1.9 trillion American Rescue Plan Act into law in March 2021, including USD 350 billion in state and local aid, billions of dollars for K-12 schools, to assist with safe reopening, vaccine research, development, distribution, etc. Furthermore, some states severely hit by the epidemic reactivated other social control measures, such as stay-at-home orders.

The fourth stage was the relaxation stage of anti-epidemic response (from May 2021 to February 2022). From May to December 2021, the Delta variant was the dominant strain of the coronavirus in the U.S. In January 2022, the Omicron variant replaced the Delta variant as the dominant strain in the U.S. At this stage, the Biden administration began to gradually lift social control measures, such as travel restrictions, and focused its anti-epidemic efforts primarily on medical control measures, such as vaccination.

In terms of medical control, the Biden administration developed an updated vaccination program, including requirements for federal employees and federal contractors to be fully vaccinated, requirements for employers with 100 or more employees to ensure their workers are fully vaccinated or tested on at least a weekly basis, and provide paid-time for employees to get vaccinated, requirements for healthcare workers at facilities participating in Medicare and Medicaid to be fully vaccinated, etc. In August 2021, the FDA approved the first COVID-19 vaccine, which was marketed as Comirnaty. In January 2022, the FDA announced the second approval of a COVID-19 vaccine, which was marketed as Spikevax. The Biden administration invested more than USD 3 billion to accelerate the discovery, development, and manufacturing of antiviral drugs. In December 2021, the FDA issued EUAs for Pfizer’s Paxlovid and Merck’s Molnupiravir for COVID-19 treatment, and AstraZeneca’s Evusheld for the pre-exposure prevention of COVID-19, respectively. Meanwhile, the Biden administration worked to ensure the supply of life-saving therapies, such as monoclonal antibody treatments, remained available for all states, and deployed federal reinforcements to support overwhelmed hospitals. In January 2022, the Biden administration asked group health plans and insurance companies to pay for at-home tests, and distributed 1 billion free at-home test kits to the country, to reach the uninsured public and those on Medicare or Medicaid. The Biden administration also provided 400 million free N95 masks to the public.

In terms of social control, in May 2021, the CDC released public health guidance stating that fully vaccinated people no longer needed to wear a mask or stay six feet away from others, except in necessary settings. In July 2021, the CDC updated guidance and recommended that fully-vaccinated people resume wearing masks indoors in areas with substantial or high transmission of COVID-19. In August 2021, the Transportation Security Administration (hereafter TSA) extended the mask-wearing requirement across all transportation networks through 18 March 2022. In October 2021, Biden signed an order imposing new vaccine requirements for air travelers, and removing severe travel restrictions on 33 countries, effective November 8. In December 2021, Biden lifted Omicron-related travel restrictions on eight countries in southern Africa. In January 2022, the Supreme Court blocked Biden’s vaccine-or-test requirements for large private companies, but allowed a vaccine mandate to stand for medical facilities that take Medicare or Medicaid payments. In February 2022, the CDC launched COVID-19 community levels (low, medium, and high) to help people and communities decide on prevention actions, i.e., layered prevention strategies.

The fifth stage was the full opening stage (from March 2022 to the present). At this stage, the dominant strains of the coronavirus in the U.S. were the Omicron subvariants BA.2, BA.5, BQ.1, BQ.1.1, XBB.1.5, and XBB.1.16, successively, with the overall mutation trend of increased transmissibility and decreased pathogenicity and infection fatality rate. In March 2022, the Biden administration released the National COVID-19 Preparedness Plan, which lifted all remaining social control measures and officially moved toward a living with COVID-19 strategy, i.e., curbing the spread of the virus and blunting its impacts only through medical control measures, rather than restricting citizens’ lives. The national emergency related to the COVID-19 pandemic terminated as President Joe Biden signed a bipartisan congressional resolution on 10 April 2023. The Department of Health and Human Services (HHS) has determined that the federal Public Health Emergency (PHE) for COVID-19, declared under Section 319 of the Public Health Service (PHS) Act, expired at the end of the day on 11 May 2023. This signals that the U.S. has turned a corner in what might be described as a pretty dark time in the fight against the pandemic.

In terms of medical control, the Biden administration established a one-stop test-to-treat system in pharmacies, clinics, and other public health settings where coronavirus tests and antiviral medications could be obtained for free. The Biden administration also launched COVID.gov, a new one-stop shop website to help people get information about lifesaving tools. Meanwhile, the Biden administration continued to distribute free at-home tests, offered the updated COVID-19 vaccines, ensured more older Americans and people with disabilities were vaccinated, and promoted booster vaccination. In December 2022, the Biden administration announced the COVID-19 Winter Preparedness Plan, including distributing more free tests to people, encouraging people to use at-home COVID-19 tests in some necessary conditions, establishing additional vaccination sites and other delivery options, close monitoring of emerging variants, etc. In April 2023, the U.S. government invested USD 5 billion in Project NextGen to advance research into new, innovative vaccines and treatments. Meanwhile, HHS stated that access to COVID-19 vaccinations and certain treatments, the FDA’s EUAs for COVID-19 products, and major telehealth flexibilities will generally not be affected, despite the end of the COVID-19 PHE. However, certain Medicare and Medicaid waivers and broad flexibilities, coverage for COVID-19 testing, and certain COVID-19 data reporting and surveillance will be affected by the end of the COVID-19 PHE.

In terms of social control, in March 2022, the TSA extended the mask-wearing requirement on public transportation and at transportation hubs through 18 April 2022, and extended it again through 3 May 2022 in April 2022. However, the TSA subsequently announced it would stop enforcing this mask mandate and implementing the directive to extend the mandate through 3 May, after a federal judge struck it down. Additionally, all of the major U.S. airlines dropped mask requirements for domestic flights. In September 2022, the CDC announced that facilities in areas without high transmission could decide whether to require doctors, patients, and visitors to wear masks at their discretion. However, some states and cities reinstated the indoor mask mandate due to the resurgence of COVID-19. In June 2022, the CDC rescinded the order requiring negative pre-departure COVID-19 test results or documentation of recovery from COVID-19 prior to flights to the U.S., but announced that proof of being fully vaccinated against COVID-19 was still required. In February 2023, the United States House of Representatives passed a bill to lift the requirement that most noncitizens show proof of being fully vaccinated against COVID-19 before entering the country. In May 2023, the Biden administration officially ended its COVID-19 vaccination requirements for federal employees, contractors, international travelers, head start educators, and CMS-certified facilities. The U.S. decided to prevent economic and educational shutdowns, and lifted almost all social control measures to stop letting COVID-19 dictate people’s lives and return America to normal routines. Moreover, the federal coronavirus response will be restructured and the coronavirus will be viewed as an endemic public health threat that can be managed through the normal authority of agencies after the end of the COVID-19 PHE.

## 4. Discussion

COVID-19 is the most serious infectious disease pandemic in the world since the 1918 influenza pandemic, presenting an unprecedented challenge to human life and economic and social development. Up to now, the COVID-19 outbreak precautions or restrictions are no longer being implemented in the vast majority of countries around the world. On 5 May 2023, the WHO announced that the COVID-19 pandemic no longer constitutes a public health emergency of international concern. As tricky as defining the beginning of the COVID-19 pandemic is, defining its end has not been easy, since there are still many related infections and deaths every day, as well as countries are also facing the threat of emerging variants. However, it is certain that the world is shifting from pandemic panic to endemic acceptance, even in China, where the outbreak first began and the long-term containment strategy was implemented. Medical control and social control are the two main tools of human response to the COVID-19 epidemic. On the one hand, in the event of infectious disease outbreaks, the U.S. has superior medical control to China, due to its advanced medical technology, abundant medical resources, and high-quality primary healthcare, and China has superior social control to the U.S., under the influence of its political system, economic structure, and cultural values. On the other hand, the differences in governance philosophy and state-society relations between China and the U.S. determine that the upper limit of what China can achieve, in terms of breadth of coverage and intensity of enforcement by adopting social control, is much higher than that of the U.S. By analyzing and summarizing the transformation nodes, different stages, control strategies, and specific measures during the COVID-19 outbreak, this study attempts to provide a general, but perhaps not exhaustive, overview of the evolution of responses to COVID-19 in China and the U.S. The major academic contribution of this study is explaining specific public health contexts in China and the U.S. that have led to different but suitable response strategies for controlling COVID-19, based on a novel perspective of “two types of control”. At the same time, a systematic review and analysis of control strategies for COVID-19 in China and the U.S. may strengthen the theoretical support for the applicability and legitimacy of the anti-epidemic mode in both countries. In addition, the theoretical modes for the prevention and control of different types of infectious disease outbreaks proposed in the paper, based on the perspective of two types of control, are theoretical advances, which are not only conducive to deepening the understanding of control strategies for different types of infectious disease outbreaks, but also provide practical guidance for the response to the next possible pandemic.

Unlike Western democracies, although officials at all levels of government in China are formally elected by the National People’s Congress (NPC) and Local People’s Congresses at all levels, the rate of electoral participation remains relatively low (in fact, the power to appoint and dismiss officials remains in the hands of the CPC, i.e., the principle of “party managing cadres”). This low rate of electoral participation inevitably leads to the central government’s concern about its legitimacy which, combined with the people-oriented culture, motivates the CPC to strive to meet people’s needs, not their wants, Wherein needs, such as survival, health, and nourishment, cannot be artificially induced by outside manipulation or satisfied symbolically, which makes them different from wants [74]. In previous normal situations, improvements in people’s living conditions have been a major source of relief from such concerns [75]. In the past three years, the ability to save more lives in comparison to Western countries has certainly been significant in consolidating legitimacy [76]. Fortunately for the Chinese government, the political system, economic structure, and cultural values have given it a strong capacity of social control. The combination of this awareness and capacity allows China to adopt stringent, extensive, and frequent social control, as well as medical control to fight the coronavirus. From the initial “zeroing” policy to the subsequent “dynamic zeroing” policy, China repeatedly achieved zero increase in indigenous confirmed or suspected cases. Thanks to superior social control, although as a country with a large gap in per capita medical resources and the level of medical technology compared to developed countries, China minimized the dramatic health consequences and avoided millions of deaths. However, the extensive, prolonged, and intense social lockdown has also led to a series of problems such as rising unemployment, declining incomes, poor access to medical care, and shortages of living materials. Even worse, although the central government has repeatedly and explicitly stated that it opposes “excessive anti-COVID measures” and “one-size-fits-all” solutions, some regions continue to adopt various excessive restrictive measures in the process of combating the epidemic, resulting in very negative policy effects and a series of tragedies which, in turn, led to heated social debates and even protests [77]. For example, in January 2022, a pregnant woman in Xi’an, Shaanxi province, was unable to be admitted to the hospital for timely diagnosis and treatment due to a lack of 48 h negative nucleic acid test report, and miscarried while waiting at the hospital gate [78]. In April 2022, during the quarantine period in Shanghai, there was a serious shortage of supplies for daily necessities, and some community workers tried to block foreign aid to maintain their single shopping channel and earn high profits [79]. In September 2022, a major traffic accident occurred in the Guizhou province involving an epidemic isolation transport vehicle transporting epidemic-involved isolates overnight for centralized isolation medical observation, resulting in 27 people being tragically killed [80]. Under the high pressure of China’s epidemic prevention and control measures, the constantly reported secondary disasters and the huge economic stress brewed more and more grievances and doubts among the population, which was greatly reinforced during the FIFA World Cup Qatar 2022 [77]. In response to these public attitudes, coupled with the fact that the virulence of the Omicron variant has waned, mass vaccination has been completed, and the treatment capability has been substantially improved, China ultimately optimized the anti-epidemic response and downgraded its management of COVID-19 by lifting almost all social control measures. This means that China has officially entered the “coexistence” stage.

Unlike China, the U.S. has one of the most mature democratic regimes on the planet. The ruling parties’ intense focus on votes means that they must carefully respond to the wants, not the needs, of the electorates, which are significantly influenced by ideologies, religiosity, and political leanings [81,82]. In a competitive setting, vote-maximizing politicians must respond to the wants of the electorates to be popular and to win elections, even at the cost of needs [74]. However, this setting greatly limits the government’s ability to impose social control in the event of an infectious disease outbreak. Although the U.S. implemented some social control measures, many potentially effective social control measures that cross the red line of democratic ideology simply cannot be enforced, due to constitutional protections of individual rights, freedoms, and privacy, as well as considerations of economic development and unemployment rates [83]. Moreover, in terms of social control, the tolerance and responsiveness of democracies to multiple voices led the U.S. to adopt a pulsed-style strategy, i.e., “relaxation–suppression–relaxation–suppression”. Specifically, when the virus is raging, the government has to respond to the wants of some elderly people with significant health preferences, or people without adequate health insurance, by implementing certain social control measures. Additionally, when the epidemic eases and the number of infections declines, the government has to respond to the reopening wants of those who are sensitive to the decrease in income, or not worried about health insurance, by lifting certain social control measures. Even worse, the U.S. is a constitution-based federal system, which means that the federal government lacks the necessary means, both in law and in executive power, to synchronize anti-epidemic policies among the states, which is precisely the core element of China’s anti-epidemic mode. To put it figuratively, while the epidemic may have just eased in New York, the situation in California, thousands of miles away, may be severe. It is as if the federal government is playing the game of “Whack-A-Mole”, and just as soon as they hold one down, another one pops up. The lack of social control has led the U.S. to rely on medical control to fight the virus. As a country with the world’s most advanced medical technology and the richest medical resources, the U.S. incorporated widespread testing, available treatments, and vaccination as the main intervention strategy, and believed it could control the epidemic sufficiently by considerably delaying or even preventing resurgence, even if social control measures were relaxed or lifted. Despite decades of preparedness work, considerable investment, and world-leading scientific expertise, the surge in COVID-19 cases placed significant strain on the U.S. healthcare system, and pushed many hospitals to breaking point, resulting in the premature death of more than one million people and unprecedented impacts on economy and society. In particular, the epidemic has highlighted and exacerbated inequalities in American society [84,85]. For example, unemployment associated with COVID-19 disproportionately affects Americans, depending on gender and race [86]. There are also racial and ethnic disparities in vaccination in the U.S. [87]. However, it should also be noted that the relentless efforts of healthcare workers, the development of several vaccines and antiviral drugs in record time, and the unprecedented vaccine push, saved countless Americans. In the meantime, the U.S. has held fast to the bottom line of the free world, in terms of protecting individual rights, freedoms, and privacy in the fight against the epidemic, even in the most difficult times, in a way that other countries, especially authoritarian ones, could hardly match.

Obscure viruses could be easily turned into epidemics or pandemics under the combined effects of climate change, antibiotic abuse, over-exploitation of nature, inadequate global public health mechanisms, and the era of globalization. For more than a millennium, human society has been watching these dramas unfold and suffering from the threats of newly emerging infectious disease outbreaks. COVID-19 is just the latest example of a devastating infectious disease outbreak. Thus, the question is not whether the next pandemic will strike, but when. Although medical control is bringing us more and more life-saving drugs, treatments, and vaccines, we cannot believe that it alone can completely overcome the threats of infectious disease outbreaks, owing to its lags and inadequacies, which provide a compelling rationale for social control. Yet, the specific measures and tools of social control still need to be deliberated and improved, due to their significant adverse impacts. All countries should draw lessons from COVID-19 and prepare for the next.

## 5. Conclusions

There are still a lot of divergent viewpoints and misunderstandings between the East and the West, particularly between the U.S. and China, when it comes to COVID-19 prevention, according to the literature, as well as from a global political and economic point of view. In an effort to create bridges between them from a scientific and philosophical perspective, this work makes a breakthrough with a specific perspective that can understand two different governance philosophy.

This paper divides national efforts to prevent and control infectious disease outbreaks into two main dimensions, i.e., medical control and social control. Based on a comparison of the differences between China and the U.S. in the two types of control, we analyze and compare the public health contexts of the two countries and provide a general, but perhaps not exhaustive, overview of the different public health priorities and the response strategies for controlling COVID-19 that result from these different contexts. By systematically reviewing and analyzing the control strategies for COVID-19 of two major countries in the world, this paper aims to strengthen the theoretical support for the scientificity and legitimacy of the two countries’ anti-epidemic modes, while attempting to provide practical guidance for the response to the next possible pandemic. Thus, this paper argues that, when faced with the same virus, different countries would adopt different response strategies, based on their endowments and limitations on “two types of control”, which may not be perfect, but must be the most suitable for the national reality.

Based on the dual views of administration and public health, this article offers a philosophical discussion and proposition. Based on these two claims, future researchers can gather more information for econometric analysis and discussion, which will help them gain a better understanding of the many forms of governance that exist in the counterpart and competition worlds of East and West, represented by China and the United States.

## Data Availability

The Global Health Security Index is available from Bloomberg School of Health Security, John Hopkins Center for Health Security (https://www.ghsindex.org/wp-content/uploads/2019/10/2019-Global-Health-Security-Index.pdf, accessed on 11 May 2023). Data and information related to the COVID-19 pandemic are available from the World Health Organization. The rest of the data involved in the article are available through the corresponding references.
